# Transcriptomic comparison of invasive bigheaded carps (*Hypophthalmichthys nobilis* and *Hypophthalmichthys molitrix*) and their hybrids

**DOI:** 10.1002/ece3.2574

**Published:** 2016-10-27

**Authors:** Jun Wang, James T. Lamer, Sarah Gaughan, Michael Wachholtz, Chenghui Wang, Guoqing Lu

**Affiliations:** ^1^ Department of BiologyUniversity of Nebraska at OmahaOmahaNE 68182USA; ^2^ Key Laboratory of Freshwater Fisheries Germplasm ResourcesMinistry of AgricultureShanghai Ocean UniversityShanghai 201306China; ^3^ Department of Biological SciencesWestern Illinois UniversityMacombIL 61455USA; ^4^ School of Interdisciplinary InformaticsUniversity of Nebraska at OmahaOmahaNE 68182USA

**Keywords:** bigheaded carps, hybridization, invasion, transcriptomes

## Abstract

Bighead carp *(Hypophthalmichthys nobilis*) and silver carp (*Hypophthalmichthys molitrix*), collectively called bigheaded carps, are invasive species in the Mississippi River Basin (MRB). Interspecific hybridization between bigheaded carps has been considered rare within their native rivers in China; however, it is prevalent in the MRB. We conducted de novo transcriptome analysis of pure and hybrid bigheaded carps and obtained 40,759 to 51,706 transcripts for pure, F_1_ hybrid, and backcross bigheaded carps. The search against protein databases resulted in 20,336–28,133 annotated transcripts (over 50% of the transcriptome) with over 13,000 transcripts mapped to 23 Gene Ontology biological processes and 127 KEGG metabolic pathways. More transcripts were detected in silver carp than in bighead carp; however, comparable numbers of transcripts were annotated. Transcriptomic variation detected between two F_1_ hybrids may indicate a potential loss of fitness in hybrids. The neighbor‐joining distance tree constructed using over 2,500 one‐to‐one orthologous sequences suggests transcriptomes could be used to infer the history of introgression and hybridization. Moreover, we detected 24,792 candidate SNPs that can be used to identify different species. The transcriptomes, orthologous sequences, and candidate SNPs obtained in this study should provide further knowledge of interspecific hybridization and introgression.

## 1 Background

Biological invasions pose serious threats to biodiversity and ecosystem integrity (Allendorf & Lundquist, 2003; Lee, 2002; Lodge, 1993). They provide unique opportunities to discern evolutionary and genetic mechanisms that underlie their ecological adaptations in novel environments (Garvey, Rettig, Stein, Lodge, & Klosiewski, 2003; Lodge et al., 1998; Sax et al., 2007). Silver carp (*Hypophthalmichthys molitrix*) and bighead carp (*Hypophthalmichthys nobilis*), collectively known as bigheaded carps, are cyprinid fishes that were introduced into the United States in the early 1970s, discovered in the wild in the early 1980s, and become established and abundant in the Mississippi River Basin (MRB) ever since (Kolar et al., 2007). The bigheaded carps outcompete some indigenous species such as gizzard shad *Dorosoma cepedianum* and bigmouth buffalo *Ictiobus cyprinellus*, attributed mainly to their trophic specialization, high mobility, rapid growth, high fecundity, and planktivorous diet (Chick & Pegg, 2001; Irons, Sass, McClelland, & Stafford, 2007; Xie & Chen, 2001), and may impact native biodiversity and local fisheries (Chick & Pegg, 2001; Irons et al., 2007; Sampson, Chick, & Pegg, 2009; Xie & Chen, 2001).

Bighead carp and silver carp diverged approximately 3.5 MYA (Tao et al., 2010). These recently diverged species can be hybridized artificially; however, natural hybridization within their native ranges is considered rare (Kolar et al., 2007; Lamer, Dolan, Petersen, Chick, & Epifanio, 2010; Yi, Liang, Yu, Lin, & He, 2006). Field surveys conducted in the MRB revealed above 20% hybridization and abundant introgression, indicating a hybrid swarm may arise in the invasive Mississippi River Basin. (Kolar et al., 2007; Lamer et al., 2010, 2014). The distributional history, hybridization, and introgression of bigheaded carps in the MRB thus offer a unique system to study adaptive radiation of a hybrid swarm of two invasive sympatric species, which would be aided by genomic resources to identify bighead carp, silver carp, and their hybrids (King, Eackles, & Chapman, 2011; Lamer et al., 2014; Mia et al., 2005).

Hybridization results in a variety of maladaptive gill raker morphologies in F_1_ hybrids, including clubbed ends, waviness, raggedness, incomplete fusion, or twists (Kipp, Cudmore, & Mandrak, 2011; Kolar et al., 2007). It was documented in bigheaded carps that possible hybrid advantages in terms of growth and fitness in the F_1_ generation might have disappeared in later generations (post‐F1 hybrids) (Green & Smitherman, 1984; Lamer et al., 2010; Voropaev, 1978). F_1_ hybrids were much less prevalent in the MRB and had a strong directional bias of silver carp maternal lineages (Lamer et al., 2010). In addition, advanced backcrosses (introgression) detected in MRB were less morphologically differentiated from the pure parental species. A functional genomic resource could facilitate evaluation of hybrid fitness.

Bighead and silver carps are different in their morphology and behavior. Bighead carp has a larger head and a dark gray coloration with irregularly shaped black splotches covering the body (Li, Wu, Wang, Chou, & Chen, 1990; Wu, 1964). The gill rakers are long and closely arranged, for general consumption of zooplankton (Conover, Simmonds, & Whalen, 2007; Jennings, 1988; Kolar et al., 2007). In comparison, silver carp has silvery sides and its gill rakers are fused into a sponge‐like apparatus for phytoplankton feeding (Conover et al., 2007; Jennings, 1988; Kolar et al., 2007). Behaviorally, silver carp is more active than bighead carp. When disturbed, silver carp leaps out of the water and may result in serious injury to boaters and anglers (Conover et al., 2007; Kolar et al., 2007). These characters may be evaluated with functional genomic approaches such as RNA sequencing (RNA‐Seq).

RNA‐Seq allows exploring genomic resources for nonmodel organisms (Du et al., 2015; Harris, O'Neill, & Munshi‐South, 2014; Stewart, Stewart, & Rivera‐Posada, 2014) and has been used to acquire transcriptomes for a number of cyprinid fishes (Gao et al., 2012; Ji et al., 2012; Liao et al., 2013; Wang, Li, Zhang, & Sun, 2012). In addition, RNA‐Seq has been successfully used to investigate transcriptomic divergence resulted from adaptive radiation to new habitats (Greenwood, Cech, & Peichel, 2012; Gross, Furterer, Carlson, & Stahl, 2013; Jeukens, Renaut, St‐Cyr, Nolte, & Bernatchez, 2010). In this study, RNA‐Seq was conducted to acquire transcriptomes of bigheaded carps and hybrids for an understanding of their invasion and hybridization in the MRB.

## 2 Materials and Methods

### 2.1 Sampling and RNA isolation

Based on gill raker morphology and other morphological characters such as body shape and color, eight bigheaded carps were collected, including two bighead carp (B1 and B2), two silver carp (S1 and S2), and four hybrids (H1, H2, H3, and H4), from the Marseilles Reach of the Illinois River (Morris, IL) in the MRB by Illinois Department of Natural Resources‐contracted commercial anglers using trammel nets of various sizes and depths (Table 1). Genotyping analysis conducted at 57 species‐specific SNPs resolved the genetic identification of the eight carps, including one pure silver carp (S2), one pure bighead carp (B2), two F_1_ hybrids (H2 and H4), and four backcross hybrids (B1, S1, H1, and H3) (Tables 1 and S1). 300–400 mg of liver tissue was biopsied using disposable 8‐mm surgical biopsy punches and transported to the laboratory on dry ice. Tissue samples were ground to a fine powder with mortar and pestle in liquid nitrogen and thoroughly mixed with varying volumes of TRIzol (Invitrogen Cat.No. 15596‐018) consistent with the manufacturer's instructions.

**Table 1:** Sampling information and RNA‐Seq summary statistics of pure and hybrid bigheaded carps in the Mississippi River Basin

Morphotype (ID)	Genotype (ID)	Sex	Raw reads	N50	Number of N50 transcripts	Mean transcript length (bp)	Largest transcript (bp)	Transcripts
Bighead carp (B1)	Second‐generation bighead carp	F	18,877,388	1,253	7,268	736	13,987	48,197
Bighead carp (B2)	Bighead carp	F	18,569,623	1,089	6,490	669	13,617	40,759
Silver carp (S1)	Second‐generation backcross with silver carp	F	16,975,045	1,187	6,669	703	13,857	43,719
Silver carp (S2)	Silver	M	19,320,499	1,221	7,329	714	13,733	46,887
Hybrid (H1)	First‐generation backcross with silver carp	M	17,676,201	1,235	7,376	724	13,990	46,898
Hybrid (H2)	F_1_ hybrid	M	19,127,407	1,232	7,364	725	15,399	47,051
Hybrid (H3)	First‐generation backcross with bighead carp	F	18,454,917	1,426	7,770	793	15,047	51,706
Hybrid (H4)	F_1_ hybrid	M	18,466,524	1,132	6,935	687	16,226	44,185

RNA was extracted from approximately 100 mg of liver tissue and purified with Qiagen RNeasy Mini Kit (Qiagen P/N 74104). Integrity of the RNA from each sample was evaluated with gel electrophoresis and absorbance readings at 260 and 280 nm (A260/280 of all samples >1.9). RNA libraries were prepared from 4 μg using the Illumina TruSeq RNA Sample Prep Kit (FC‐122‐1002) following the manufacturer's instructions. Two indexed libraries of all the eight samples were pooled together and sequenced on the Illumina Genome Analyzer IIx using two 36‐cycle sequencing kits to read 76 nucleotides (nt) from a single end of each insert, via standard multiplexing v 8.3 protocol.

### 2.2 Transcriptome sequencing reads cleaning, assembly, and annotation

Reads were quality‐filtered using Trimmomatic read trimming tool (Bolger, Lohse, & Usadel, 2014). Reads containing 3′ or 5′ ends with an average quality score below 20 in a 4‐base pair sliding window were trimmed, and those below 5 at the beginning and from the end also were trimmed (Bolger et al., 2014). Any reads shorter than 53 nt were excluded for further assembly on Trinity version 2012‐06‐08 (Grabherr et al., 2011) with the single‐end mode under default parameters. The protein coding sequence (CDS) was predicted by TransDecoder implemented in Trinity with a minimum length of 100 amino acids. The assembled transcripts then were annotated using BLASTX against NCBI‐NR, UniProt protein databases, and Ensembl zebrafish protein sequences (Zv9; Flicek et al., 2014, UniProt Consortium, 2014), with a cutoff *E*‐value less than 1 × 10^−10^. Gene Ontology (GO) and KEGG analyses were performed with the BLAST2GO program (Conesa et al., 2005). Unigene IDs were assigned to the assembled transcripts based on the best match to Ensembl zebrafish proteins (Zv9).

### 2.3 Orthologous gene identification and neighbor‐joining tree construction

The longest protein sequence from each gene was used to identify clusters of orthologous gene groups. A pairwise BLAST was conducted between every two proteomes (whole protein sets) with InParanoid 4.1 (Östlund et al., 2010), and all pairwise orthologs were combined into orthologous groups using MultiParanoid (Alexeyenko, Tamas, Liu, & Sonnhammer, 2006). All one‐to‐one orthologous groups were assigned a cluster ID for further analysis. A PERL script was written to extract the orthologs. The protein sequences of the orthologs were concatenated and aligned using MAFFT (Katoh, Misawa, Kuma, & Miyata, 2002). A neighbor‐joining (NJ) tree with Jones–Taylor–Thornton model and 1,000 bootstrapping replicates was used to elucidate the relationship among the samples using MEGA 5.0 (Tamura et al., 2011). The Ka/Ks ratio, nonsynonymous changes per nonsynonymous site (Ka) to the synonymous changes per synonymous site (Ks), was calculated to test positive selection on these orthologous genes using PAML with the yn00 algorithm (Yang, 2007).

### 2.4 Identification of species‐specific SNPs

The raw reads from silver and bighead carps were mapped to the silver carp transcriptome using bwa (v 0.7.9a‐r786) with the bwa mem algorithm (Li & Durbin, 2010). SNPs were called using SAMtools (v 0.1.18) (Li et al., 2009). Candidate species‐specific SNPs for bighead and silver carps were filtered according to the following criteria: read depths >10, a minimum quality score of 20, and homozygous in either species.

## 3 Results

### 3.1 Transcriptome assembly and annotation

A total of approximately 147 million sequencing reads were generated, ranging from ~17.0 million reads in second‐generation backcross silver carp (S1) to ~19.3 million reads in pure silver carp (S2) through RNA‐Seq of eight individuals of bigheaded carps (Table 1). After filtering low‐quality reads, 40,759–51,706 transcripts were assembled. The bighead carp (B2) had the lowest N50 (length of longest contig that has at least 50% match with all of the other contigs of the assembly), smallest number of N50 transcripts, and shortest mean transcript length, whereas the first‐generation backcross with bighead carp (H3) had the highest N50, largest number of N50 transcripts, and longest mean transcript length. The largest transcript ranged from 13,617 (B2) to 16,226 (H4).

Pure bighead carp (B2) had the lowest number of transcripts (40,759), whereas the first‐generation backcross with bighead carp (H3) had the highest (51,706). More transcripts were found in silver carp (S2) (46,887) than in bighead carp (B2) (40,759). One F_1_ hybrid (H2) had more transcripts than parental species, whereas another F_1_ hybrid (H4) had slightly above their average. For the annotated unigenes, 8,665 of them were shared between bighead and silver carps, which account for 88.0% and 83.7% of their transcriptomes, respectively.

BLAST searches against three protein databases (NCBI‐NR, UniProt, and Ensmeble zebrafish Zv9) resulted in comparable number of transcripts with significant hits and over 50% of the transcriptomes annotated for each individual. The number of transcripts annotated based upon zebrafish proteins (Zv9) ranged from 22,993 (B2) to 27,914 (H3). Variable numbers of annotated transcripts were observed between two F_1_ hybrids (H2, H4) as well as between two first‐generation backcrosses (H3 and H1). The number of predicated protein CDS with minimum nucleotide length of 300 bp was 15,588 (B2) to 21,336 (H3) (Table 2). Similarly, variation in the number of CDS occurred between two F_1_ hybrids as well as between two first‐generation backcrosses.

**Table 2:** Protein and CDS predication of transcriptomic sequences of carp samples, with the number of transcripts mapped to Gene Ontology (OG) and KEGG pathways

Morphotype ID	Number of annotated transcripts			Number of CDS (>300 bp)	Gene Ontology	KEGG
	NCBI‐NR	UniProt	Ensembl Zebrafish			
B1	26,552	26,466	26,227	19,106	17,749	15,479
B2	23,069	22,179	22,993	15,588	15,756	13,838
S1	24,196	20,336	23,712	16,531	16,747	14,747
S2	25,235	21,127	25,070	17,600	17,404	15,280
H1	25,513	25,246	25,196	17,896	17,619	15,344
H2	25,689	21,540	25,535	18,252	17,647	15,320
H3	28,133	23,701	27,914	21,336	19,084	16,596
H4	24,597	20,617	24,270	16,703	16,954	14,884

**Figure 1:**
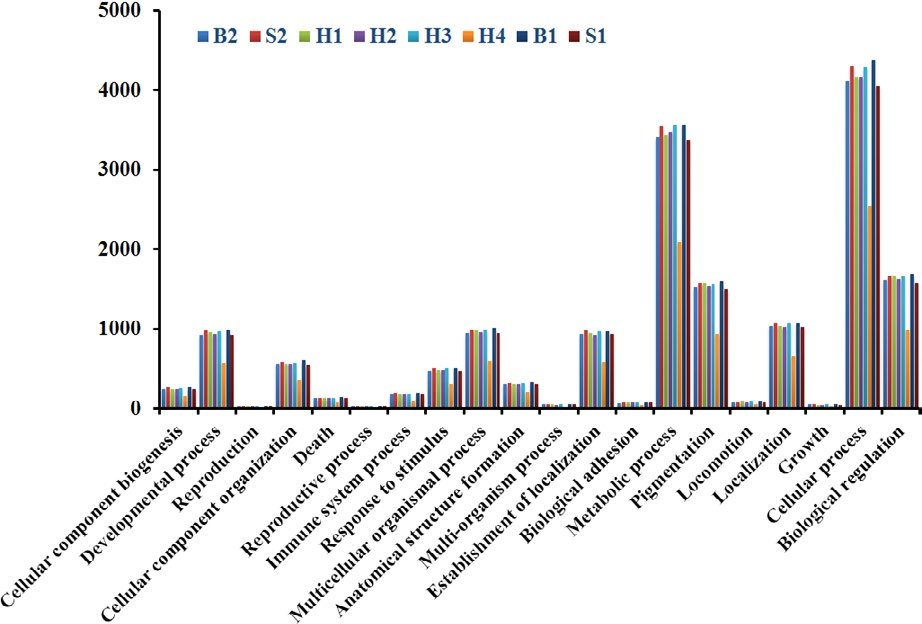
The number of transcripts found in each Gene Ontology Biological Processes category in pure bighead carp (B2), pure silver carp (S2), F_1_ hybrids (H2 and H4), and backcrosses (B1: 2nd‐gen bighead carp, H1: 1st‐gen silver carp, H3: 1st‐gen bighead carp, and S1: 2nd‐gen silver carp). One F1 hybrid (H4), when compared with other bigheaded carp samples, was found with a less number of transcripts in most categories

Further GO analysis showed that 15,756 to 19,084 transcripts successfully mapped to 23 GO biological processes (Figure 1). Most transcripts were related to both cellular and metabolic processes. Comparable numbers were identified among all carp samples except H4. Although both H2 and H4 are male F_1_ hybrids of silver carp (♀) × bighead carp (♂) according to the SNP genotyping, the number of transcripts in each category was lower in H4 compared to H2. A total of 13,838–16,596 transcripts were mapped to 127 KEGG pathways, including 124 for pure bighead carp (B2) and 122 for pure silver carp (S2) (Table 2). Of the top 10 KEGG pathways, purine metabolism, pyrimidine metabolism, tryptophan metabolism, pyruvate metabolism, glycolysis/gluconeogenesis, glycine, serine, and threonine metabolism are common to both species. Both species had seven enzymes found in the oxidative phosphorylation pathway; however, more transcripts were detected in silver carp. For the glutathione metabolism pathway (related to detoxification), more enzymes were found in silver carp (16) than in bighead carp (14). F_1_ hybrids, H2 and H4 had an equivalent number of enzymes in most pathways, however, varied in tryptophan metabolism, starch and sucrose metabolism, and steroid hormone biosynthesis pathways (Table S2).

### 3.2 Transcriptomic comparison among bigheaded carps and hybrids

**Figure 2:**
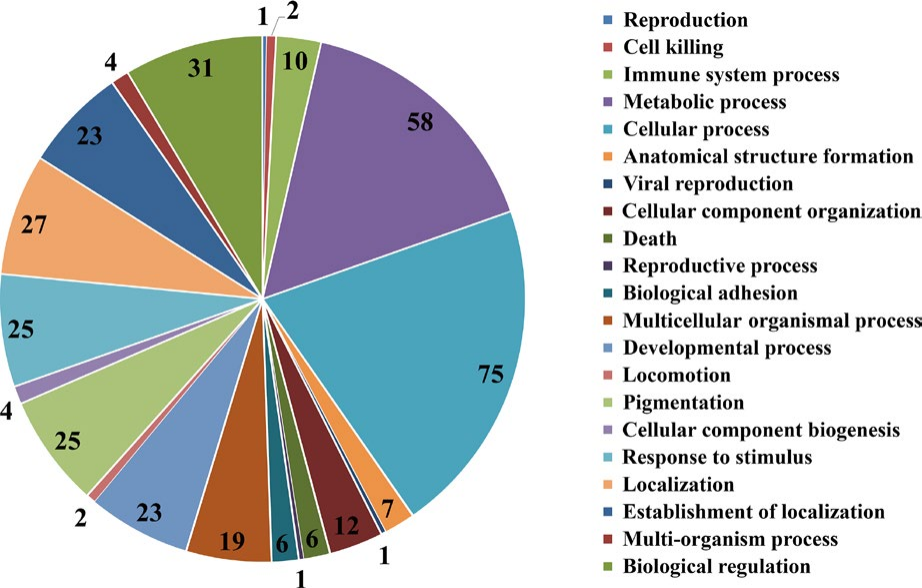
Distribution of the number of genes under positive selection that were mapped to main Gene Ontology Biological Processes categories in bigheaded carps

We identified 7,007 pairs of one‐to‐one orthologs between bighead and silver carps (Table S3). A total of 230 candidate genes appeared under positive selection (Ka/Ks > 1) (Table S4). Further GO analysis revealed 10 in GO:0002376 (immune system process), 23 in GO:0032502 (developmental process), and 25 in GO:0050896 (response to stimulus) (Figure 2). We found positive selected genes associated with response to hypoxia or stress such as *cox4i1*,* ppiab*, and *hspb8* or linked to immune response such as *mhc1zba*,* cd74b*, and *cfp* (Table S4).

**Figure 3:**
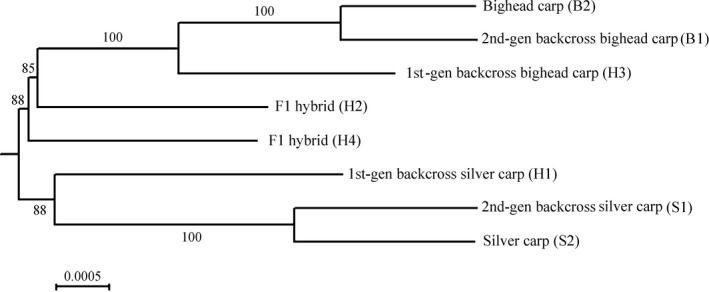
Neighbor‐joining tree demonstrating the relationship among pure and hybrid bigheaded carps. The sample names are composed of genotypes based on SNP analysis, species name and morphotypes based on morphological characters including gill rakers (in parentheses). Scale represents the amino acid substitution rate

Among eight transcriptomes, 2,514 pairs of one‐to‐one orthologs were identified (Table S5). The NJ tree constructed with concatenated sequences of all orthologs depicted the B1 (second‐generation backcross hybrid) clustered closer to B2 (bighead carp). Genotyping analysis of 57 species‐specific SNPs developed by Lamer et al. (2014) identified B1 as a hybrid; however, transcriptomic data suggested B1 was a second‐generation backcross with bighead carp (Figure 3). A similar situation occurred between S1 (second‐generation backcross with silver carp) and S2 (pure silver carp), whose transcriptomic analysis suggests S1 should be a post‐first‐generation backcross with silver carp. The NJ tree revealed H2 and H4 located toward the middle of the tree, corresponding to SNP genotyping result. H1 and H3 were originally identified as hybrids based on morphology. Our transcriptomic tree showed that H1 and H3 were first‐generation backcross with silver carp and first‐generation backcross with bighead carp (Table 2). The NJ tree based upon over 2,500 orthologous sequences demonstrated high resolution of a transcriptomic approach in uncovering the complex evolutionary relationships among bigheaded carps within a hybrid swarm.

### 3.3 Identification of candidate species‐specific SNPs

A total of 24,792 candidate SNPs were identified to be species specific, which can be used as markers for the identification of bighead carp or silver carp. Among these SNPs, 19,511 candidate SNPs could be annotated by 3,643 genes (Table S6).

## 4 Discussion

We described the transcriptomes of bigheaded carps sampled in the MRB. The number of annotated transcripts was found slightly different but comparable. Over 80% of the annotated unigenes were shared, and similar number of GO annotations was identified between bigheaded carps, indicating high transcriptomic similarity, which may explain why bighead carps can hybridize in the MRB. Transcriptomic variation, however, was also identified, which may to some extent reflect the biological functions of the two species as well as different sexes. More transcripts were detected in silver carp for the oxidative phosphorylation pathway, which could reflect the hypersensitive nature of this leaping fish, and more enzymes in glutathione metabolism pathway related to detoxification were found in silver carp than in bighead carp, indicating that silver carp may require more enzymes to detoxify algae (Kipp et al., 2011; Kolar et al., 2007). In addition, more transcripts were assembled in first‐generation backcross with bighead carp (H3) than pure bighead carp (B2), indicating novel transcripts may be generated through more generations of hybridization, which may cause another ecological concern related to the fitness and spread of hybrids in the MRB (Kolar et al., 2007; Lamer et al., 2010).

Analysis of both invasive and native silver carp yielded a similar number of annotated transcripts, but differed in total number (Fu & He, 2012). The difference between the total number of transcripts in native and invasive silver carp could be attributed to the number of tissues used for analysis and/or the number of raw reads produced. The transcriptomes of several other cyprinid species have been reported, including common carp, crucian carp, blunt snout bream (*Megalobrama amblycephala*), and many other species, and the number of annotated transcripts among them including bigheaded carps ranges from 22,000 to 25,000, except for blunt snout bream (Gao et al., 2012; Liao et al., 2013; Wang et al., 2012). Transcriptomes are known to vary in different tissues, developmental stages, and physiological conditions. Comparison of transcriptomes from different studies, even for the same species, merits caution. Previous study indicated response to stimulus (GO: 0050896) was overrepresented in silver carp compared to zebrafish (Fu & He, 2012) and in our study, more transcripts were also annotated with response to stimulus in silver carp than bighead carp, which may also reflect the hypersensitive nature of silver carp.

Transcriptome is a useful resource that can be used to identify orthologous genes for the inference of evolutionary relationship in a number of aquatic species. The characterization of orthologous genes between common carp and zebrafish transcriptome allowed the evaluation of the fourth round of genome duplication in common carp (Wang et al., 2012). The phylogeny inferred with the orthologous genes derived from the transcriptomes of two eastern African cichlid species, *Astatotilapia burtoni* and *Ophthalmotilapia ventralis,* revealed a clear evolutionary relationship between the two species (Baldo, Santos, & Salzburger, 2011). The transcriptomic comparison of two young, sympatric sister species, benthic *Amphilophus astorquii* and limnetic *Amphilophus zaliosus,* resulted in over 13,000 orthologs and revealed 6 genes under strong positive selection (Elmer et al., 2010). In this study, we identified 2,500 one‐to‐one orthologous gene sequences which will provide a valuable resource for evolutionary study in future work. Meantime, our NJ tree based upon over 2,500 orthologous gene sequences demonstrated a high resolution of the transcriptomic approach in uncovering the complex evolutionary relationships among bigheaded carps within a hybrid swarm.

The 230 candidate positively selected genes identified in this study may reflect the adaptive evolution of bigheaded carps during speciation (Tao et al., 2010). Immune‐related genes were usually strongly selected during evolution and were identified as selected during teleost fish evolution (Eizaguirre, Lenz, Kalbe, & Milinski, 2012; Malmstrom et al., 2016; Xiao et al., 2015). In this study, a number of immune‐related genes were detected under positive selection, which may reflect the rapid evolution of bigheaded carps. Although bighead carp and silver carp look morphologically similar, behavioral and physiological differences do exist: silver carp is more sensitive to disturbance, whereas bighead carp is more resistant to hypoxia (Conover et al., 2007; Jennings, 1988; Kolar et al., 2007). Accordingly, we identified positively selected genes such as *cox4i1*,* ppiab*, and *hspb8* that are associated with stress responses.

Large variation in the transcriptomes and their functional categories was identified in two F_1_ hybrids, with one F_1_ hybrid (H4) possessing a lower number of transcripts in all biological processes categories, which may suggest lower fitness in H4 compared to another hybrid (H2). Variation in fitness is expected in F_1_ hybrids and may affect growth, survival rates, and hybrid vigor (Abbo & Ladizinsky, 1994; Rosas, Barton, Copsey, Barbier De Reuille, & Coen, 2010). Any benefits or detriments to hybridization might affect differential relative fitness of individuals (Campbell & Waser, 2007; Seehausen, 2004). F_1_ individuals were not prevalent in the MRB and individuals were morphologically deformed, which implies that selection most likely acts against F_1_ hybrids (Kolar et al., 2007; Lamer et al., 2010). However, the issue pertaining to F_1_ hybrid fitness in bigheaded carps requires further investigation with larger sample sizes and using an integrated approach of genomics, morphology, behavior, etc.

Hybridization is more common in fish than other vertebrates (Leary, Allendorf, & Sage, 1995). Most fish exhibit external fertilization and similar mating behaviors, which may facilitate interspecific hybridization. Bigheaded carps are rarely known to hybridize within their native river systems. Within the native ranges, reproduction isolation may be maintained by spatial or temporal isolation rather than pre‐ or postzygotic barriers (Mayr, 1970). This study provides an insight into hybridization and introgression of bigheaded carps, which supports the prediction that bigheaded carps in the MRB are likely to form a hybrid swarm (Lamer et al., 2014). This introgressive hybridization may result from a shift to a more homogenous reproductive environment in the MRB which lacks extrinsic factors that are required for the restriction of gene flow between species (Seehausen, Takimoto, Roy, & Jokela, 2008).

## 5 Conclusions

We obtained multiple transcriptomes of pure, F_1_ hybrids, and backcrosses of bigheaded carps. We found variation between two F_1_ hybrids that may indicate potential hybrid inferiority. The transcriptomic comparison offers a means for elucidating relationships among pure and hybrid bigheaded carps. The transcriptomic repository will facilitate insights into genomic introgression and hybridization and lay a foundation for future studies.

## Conflict of Interests

The authors declare that they have no competing interests.

## Available Supporting Data

Data for this study are available at NCBI SRA database with the following accession numbers: SRR3224768, SRR3224767, SRR3224766, SRR3224765, SRR3224687, SRR3224771, SRR3224770, and SRR3224769.
